# ResBoost: characterizing and predicting catalytic residues in enzymes

**DOI:** 10.1186/1471-2105-10-197

**Published:** 2009-06-27

**Authors:** Ron Alterovitz, Aaron Arvey, Sriram Sankararaman, Carolina Dallett, Yoav Freund, Kimmen Sjölander

**Affiliations:** 1 Department of Computer Science, University of North Carolina at Chapel Hill, USA; 2 Department of Computer Science and Engineering, University of California, San Diego, USA; 3 Department of Electrical Engineering and Computer Sciences, University of California, Berkeley, USA; 4 Department of Bioengineering, University of California, Berkeley, USA

## Abstract

**Background:**

Identifying the catalytic residues in enzymes can aid in understanding the molecular basis of an enzyme's function and has significant implications for designing new drugs, identifying genetic disorders, and engineering proteins with novel functions. Since experimentally determining catalytic sites is expensive, better computational methods for identifying catalytic residues are needed.

**Results:**

We propose ResBoost, a new computational method to learn characteristics of catalytic residues. The method effectively selects and combines rules of thumb into a simple, easily interpretable logical expression that can be used for prediction. We formally define the rules of thumb that are often used to narrow the list of candidate residues, including residue evolutionary conservation, 3D clustering, solvent accessibility, and hydrophilicity. ResBoost builds on two methods from machine learning, the AdaBoost algorithm and Alternating Decision Trees, and provides precise control over the inherent trade-off between sensitivity and specificity. We evaluated ResBoost using cross-validation on a dataset of 100 enzymes from the hand-curated Catalytic Site Atlas (CSA).

**Conclusion:**

ResBoost achieved 85% sensitivity for a 9.8% false positive rate and 73% sensitivity for a 5.7% false positive rate. ResBoost reduces the number of false positives by up to 56% compared to the use of evolutionary conservation scoring alone. We also illustrate the ability of ResBoost to identify recently validated catalytic residues not listed in the CSA.

## Background

A tenet of modern molecular biology is that protein structure determines function. However, only a small subset of a protein's residues are required for the protein to perform its function [1]. Identifying these critical residues is crucial to understanding the molecular basis of a protein's function and has significant implications for designing new drugs, identifying genetic disorders, and engineering proteins with novel functions [2,3].

The goal of catalytic residue prediction is to identify the set of residues that are directly involved in the biochemical reaction performed by an enzyme. Porter et al. [4] define catalytic residues as those that (1) have direct involvement in the catalytic mechanism, e.g. as a nucleophile, (2) alter the pK_a _of a residue or water molecule directly involved in the catalytic mechanism, (3) stabilize a transition state or intermediate, thereby lowering the activation energy for a reaction, or (4) activate the substrate in some way, e.g. by polarizing a bond to be broken.

Traditional biochemical methods to identify catalytic residues require extensive experimentation-mutagenesis experiments followed by exhaustive testing of the enzyme's catalytic performance, including concentration assays. These experiments are time-consuming and expensive, especially when applied to large numbers of putative residues. In addition, accurate assays of a protein's assumed function must be available. Given the difficulty of determining catalytic residues experimentally, computational tools to assist in the prediction of catalytic residues are crucial.

Our method aims to encode intuition into a computational framework. To narrow the list of candidate residues for mutagenesis, experimentalists often rely on their experience, using rules of thumb such as: catalytic residues are evolutionarily conserved, solvent accessible, found in clusters, typically hydrophilic, and located inside pockets. Each of these rules of thumb is based on a reasoned, previously tested, underlying biological hypothesis. However, a single rule cannot fully explain the variety of catalytic residues produced by billions of years of evolution. As discussed in the Results and Discussion sections, identifying catalytic residues computationally without introducing large numbers of false positives has proved challenging, particularly for methods that depend on only one or two properties of a catalytic site.

In this paper, we propose a new method, ResBoost, to computationally learn characteristics of catalytic residues. The method effectively selects and combines rules of thumb into a single simple, easily interpretable logical expression that can be used for prediction. We formally define rules of thumb for catalytic residue prediction based on evolutionary conservation, 3D clustering, hydrophobicity, solvent accessibility, pocket accessibility, catalytic propensity, and secondary structure. Our method then builds on the machine learning algorithm AdaBoost and Alternating Decision Trees to generate logical expressions.

**Figure 1:**
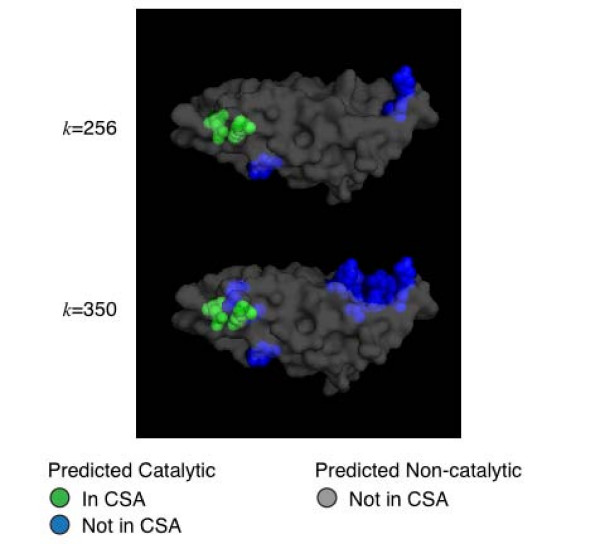
**We demonstrate ResBoost's control over the sensitivity/specificity trade-off using the enzyme 7,8-dihydroneopterin aldolase, a bacterial and plant enzyme needed for folate production that is an important target for antibiotics **[28]. ResBoost predictions for this enzyme from *Staphylococcus aureus *(PDB ID: 2dhn) for two values of the trade-off parameter *k*, *k *= 256 (top) and *k *= 350 (bottom), are shown. ResBoost detected the main reaction center E22 and K100. In addition, at *k *= 350, ResBoost detected another cleft that includes Y54, a newly discovered catalytic residue not yet in the CSA that has been found to be important in orienting the substrate and stabilizing the intermediate.

All protein quantitative data is inherently noisy, thus any method to predict catalytic residues is subject to an inherent trade-off between sensitivity (the number of correct catalytic residue predictions relative to the total number of catalytic residues) and specificity (the number of residues correctly identified as non-catalytic relative to the total number of non-catalytic residues). We provide the user with control over this trade-off: the user specifies an input parameter *k *and the method maximizes sensitivity while maintaining the desired specificity (or false positive rate (FPR)). The result for an example enzyme, 7,8-dihydroneopterin aldolase from *Staphylococcus aureus *(PDB ID: 2dhn), is shown in Figure 1.

Several computational approaches have been previously developed to help identify catalytic residues as well as other functionally important residues in proteins. Early methods exploited information from sequence only. Casari et al. developed one of the first computational approaches by considering protein sequences in a multiple sequence alignment (MSA) to be points in a high-dimensional space and using Principal Component Analysis to predict specificity positions [5]. In developing the Evolutionary Trace (ET) method, Lichtarge et al. incorporated the use of phylogenetic analysis of homologous proteins in the prediction of functional residues [6,7]. The ET method, which also requires an MSA as input, is based on the assumption that functional residues are conserved during evolution relative to other residues. The original method [6] scores residues by their evolutionary importance by cutting a phylogenetic tree based on a partition identity cutoff. Subsequent extensions to this method included entropy-based approaches to improve specificity and sensitivity [8]. The ConSurf algorithm estimates the rate of evolution of each residue of the protein from the sequence and phylogenetic information, and then maps these rates onto the molecular surface of the protein to help identify patches that may be functionally important [9,10]. The SequenceSpace Automatization Method reduces manual intervention in the method proposed by Casari et al. [11]. Extensions of ET have been proposed that cluster the predicted functional residues [12] and use the variability of an alignment column in addition to the trace values [8]. Mayrose et al. [13] extended ConSurf to use empirical Bayesian methods to measure the evolutionary rate. Glaser et al. [14] used ConSurf to refine ligand-binding pocket predictions. Sankararaman and Sjölander introduced INTREPID, a novel catalytic site prediction algorithm using sequence information only which uses a phylogenetic tree traversal and Jensen-Shannon divergence to identify the most informative point in a phylogenetic tree for each position in a sequence, enabling their method to make use of highly divergent homologues [15]. Recent methods have begun to explicitly integrate information from a protein's tertiary structure when ranking residues [16-19]. Methods exploiting multiple sources of information include those using neural networks [20,21] and Support Vector Machines (SVM's) [19,22-24]. We discuss these various approaches in greater detail in the Discussion.

The approach we develop in this paper aims to characterize catalytic residues by introducing simple logical expressions that are transparent and intuitive. ResBoost combines multiple predictors of catalytic residues into a single, easily-interpreted classifier that simultaneously provides explicit user control over the trade-off between sensitivity and specificity.

Our method uses AdaBoost to iteratively sets weights for the rules of thumb, or base classifiers, and adds them as leaves to an Alternating Decision Tree data structure during a training phase to minimize the number of prediction errors for a training set. With each iteration of the training phase, AdaBoost treats each residue asymmetrically, placing more weight on incorrectly predicted residues in order to reduce the training error at an exponential rate. The algorithm produces a tree data structure containing a weighted base classifier at each leaf, enabling new residues to be classified by summing weights along all feasible branches of the tree. We simplify the logic behind the Alternating Decision Tree to create a compact logical expression composed of rules of thumb that identifies catalytic residues, enabling the intuitive classification of residues not previously seen by the method (i.e. not in the training set).

To learn the characteristics of catalytic residues and evaluate our prediction results, we use a data set composed of enzymes available in the Catalytic Site Atlas (CSA) [4], an online database that provides annotations for enzymes in the Protein Data Bank (PDB). The CSA annotations specify catalytic residues that have been experimentally validated and published in the primary literature. Our data set is a randomly selected subset of 100 evolutionarily divergent enzymes from the CSA. We use 10-fold cross-validation with distinct training and evaluation data sets to provide an accurate picture of how our method will perform on new enzymes submitted for analysis in the future.

## Results

### Characterizing catalytic residues

ResBoost characterizes catalytic residues using a logical expression of simple rules. The logical expression is computationally learned during the method's training phase. As discussed in the Methods section, the parameter *k *provides precise control over the trade-off between sensitivity and specificity. Increasing *k *increases the importance of sensitivity relative to specificity during the training phase. For different trade-off values *k*, ResBoost computes different logical expressions for characterizing catalytic residues. Once training is complete, ResBoost can be applied to new enzymes by evaluating the learned compact logical expression for the desired sensitivity/specificity trade-off *k*. We write each logical expression below in disjunctive normal form [25], which consists of a disjunction of conjunctive clauses of the rules. For instance, there are two clauses for *k *= 256 below, and a residue is classified as catalytic if both or either one is true.

For *k *= 256, a residue is classified as catalytic if any of the following is true:

1. in a cluster AND not hydrophobic, OR

2. in a cluster AND in a pocket with solvent accessible surface area > 35.36 Å^2 ^AND has global conservation score > 0.9.

For *k *= 128, a residue is classified as catalytic if any of the following is true:

1. in a cluster AND not hydrophobic AND has global conservation score > 0.9, OR

2. in a cluster AND not hydrophobic AND in a pocket with solvent accessible surface area > 35.36 Å^2^, OR

3. in a cluster AND in a pocket with solvent accessible surface area > 35.36 Å^2 ^AND has global conservation score > 0.9.

For *k *= 64, a residue is classified as catalytic if any of the following is true:

1. in a cluster AND not hydrophobic AND has global conservation score > 0.9, OR

2. in a cluster AND not hydrophobic AND in a pocket with solvent accessible surface area > 35.36 Å^2^.

For higher *k*, the resulting logical expression is broader, enabling the classifier to be more inclusive and achieve higher sensitivity. As *k *is decreased, the logical expression becomes narrower (stricter and/or fewer conjunctions) to reduce false positives. For example, as *k *is decreased from 128 to 64, the third conjunction of rules is removed, reducing the likelihood of false positives.

**Figure 2:**
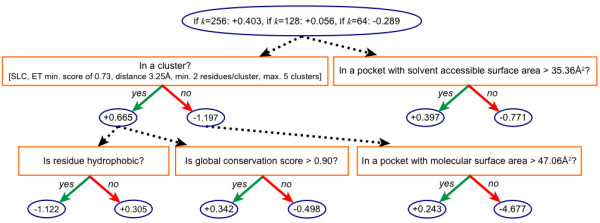
**Computed ATrees for ResBoost for *k *= 256, 128, and 64**. A residue is predicted as catalytic if its final score determined by the ATree is greater than 0. To evaluate the score for a particular residue, start at the root circle at the top of the tree, which specifies an initial residue score. Follow each branch (dotted lines) from the circle to each question box, which is based on a specific base classifier. From each box, follow the edge with the correct answer (solid line) to the next circle, adding the value in the circle to the residue score. Continue until each branch (dotted line) that is reached is followed.

As described in the Methods section, the logical expressions were computed automatically in the training phase of ResBoost. We illustrate in Figure 2 the ATrees generated during training for three values of *k *for a randomly selected fold from the cross-validation (described below), which is representative of the final classifier when applied to the entire dataset. To read the tree for a particular residue, start at the root circle at the top of the tree. The circle at the top of the tree specifies an initial score for the residue. Follow each branch (dotted line) from the circle to each question box, which is based on a specific base classifier. From each box, follow the edge with the correct answer (solid line) to the next circle, adding the value in the circle to the residue score. Continue adding scores along until each branch (dotted line) to compute a final score. If the final score is positive, the residue is predicted as catalytic.

The only significant difference between the ATrees for *k *= 256, 128, and 64 is the starting value in their root. As shown by the logical simplifications above, the 5th base classifier (bottom right) had no effect on the final classification. As described by the cross-validation results below, ResBoost achieves high quality results with just four well-chosen rules of thumb.

### Cross-validation results

**Figure 3:**
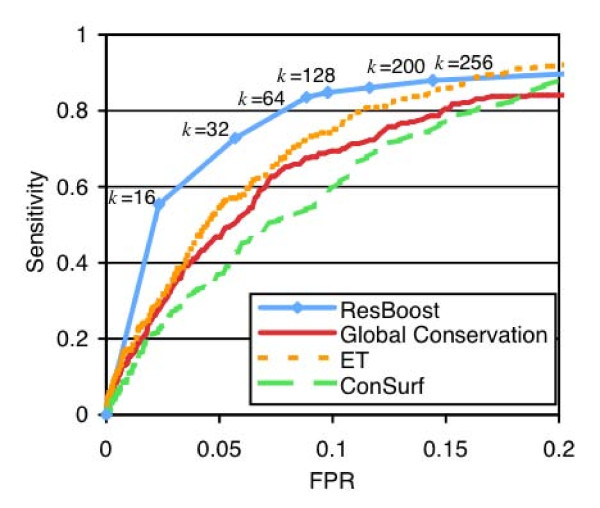
**Sensitivity vs. false-positive rate curve for ResBoost compared to Global Conservation, ET, and ConSurf based on normalized score thresholding**. At 85% sensitivity, ResBoost cuts the false positive rate by 55% compared to global conservation, 48% compared to ConSurf, and 32% compared to ET.

Figure 3 illustrates the Receiver-Operator Characteristic (ROC) curve indicating the trade-off between sensitivity and FPR for ResBoost for increasing values of *k*. As described in the Methods section, for methods that generate residue scores rather than classifications, we normalized all scores for each score type on a protein by protein basis and then used a score threshold to classify residues as catalytic or non-catalytic. Using the CSA as ground truth, ResBoost achieved a sensitivity of 73% at a false positive rate of 5.7% (*k *= 32), a sensitivity of 85% at a false positive rate of 9.8% (*k *= 128), and a sensitivity of 88% at a false positive rate of 14% (*k *= 256). Increasing *k *improves sensitivity but also introduces more false positives.

**Figure 4:**
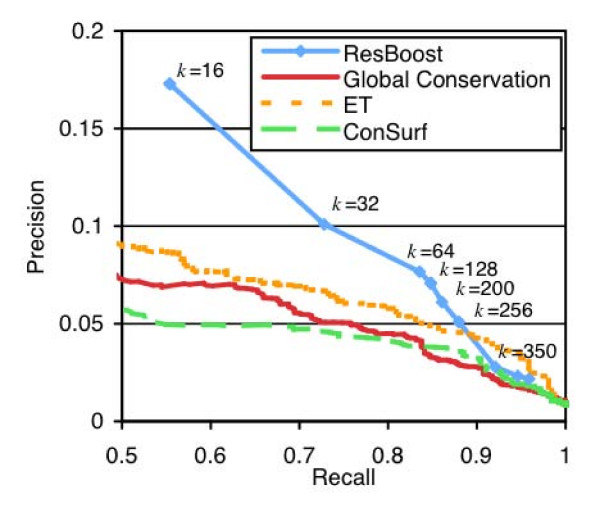
**Precision vs. recall curves for ResBoost compared to Global Conservation, ET, and ConSurf based on normalized score thresholding**. At a recall of 73%, ResBoost improves precision by 98% compared to global conservation, 120% compared to ConSurf, and 51% compared to ET.

Figure 4 illustrates the trade-off between precision and recall for ResBoost for increasing values of *k*. Based on the CSA classifications, ResBoost achieved a precision of 17% at a recall of 55% (*k *= 16), a precision of 7.1% at a recall of 85% (*k *= 128), and a precision of 5.1% at a recall of 88% (*k *= 256). Increasing *k *improves recall but decreases precision.

We also compare ResBoost with the results of score thresholding for global conservation, ET, and ConSurf. As shown in Figure 3, ResBoost increases sensitivity at low false positive rates. At a false positive rate of 5.7%, ResBoost achieved sensitivity of 73% compared to 51% for global conservation, 57% for ET, and 43% for ConSurf. At a false positive rate of 9.8%, ResBoost achieved sensitivity of 85% compared to 69% for global conservation, 74% for ET, and 59% for ConSurf.

### Analysis of specific ResBoost predictions

We analyze predictions made by ResBoost on two example enzymes: scytalone dehydratase (PDB ID: 1std) and dihydroneopterin aldolase (PDB ID: 2dhn). To illustrate our results, we used the ResBoost classifier obtained during cross-validation for the fold in which these enzymes were in the test set, i.e., not used to train the method.

Our first example is scytalone dehydratase (PDB ID: 1std) in the pathogenic fungus *Magnaporthe grisea*. This enzyme is a lyase, catalyzing the dehydration of scytalone and vermelone – a required step in the pathogenesis of the fungus to commercial rice. Scytalone dehydratase is the target of fungicides which release a synthetic inhibitor of the enzyme (Carpropamid), thereby inhibiting fungal infection [26]. Ligand-bound structures and mutational studies have found seven residues to be important and part of the catalytic site of this enzyme: Asn131, Asp31, His85, His110, Ser129, Tyr30, and Tyr50 [26]. These residues are important for the syn elimination mechanism; Ser129 helps orient the substrate within the active site, His85 acts as a general base and a general acid in the syn elimination, and Tyr30 and Tyr50 act in protonating the substrate's carbonyl group through a water molecule. Of these seven residues, all except the Ser129 and Asn131 are listed in the CSA entry for 1std.

**Figure 5:**
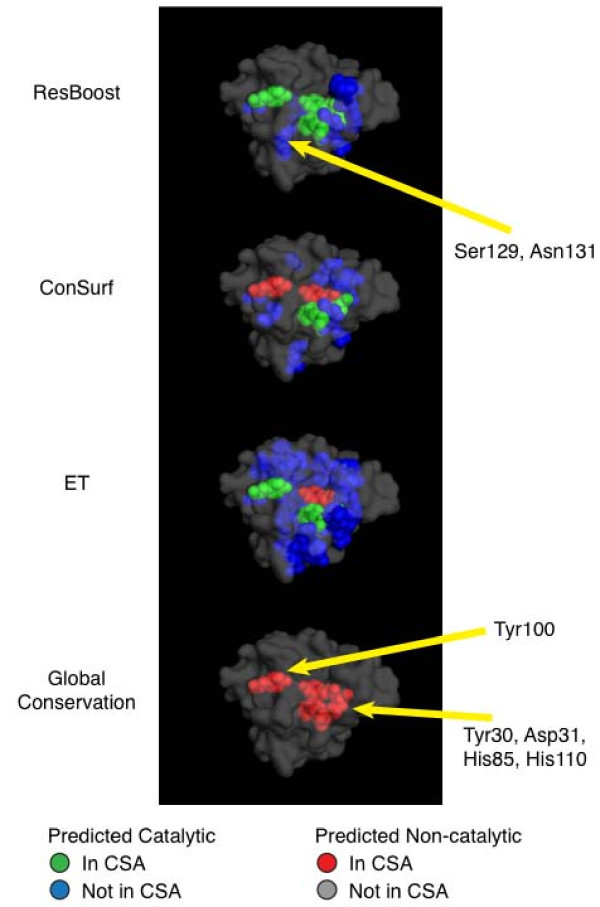
**Comparison of ResBoost, ConSurf score thresholding, ET score thresholding, and global conservation methods on *Magnaporthe grisea *scytalone dehydratase **(PDB ID: 1std). For a fixed specificity, ResBoost is more sensitive than ConSurf, ET, and global conservation. The tradeoff parameter *k *for ResBoost is set to 128. ResBoost predicts all residues listed as catalytic in the Catalytic Site Atlas (CSA) i.e., Tyr30, Asp31, Tyr50, His85, and His110. ConSurf correctly predicts four catalytic residues (Asp31, Tyr50, His85, and His110), ET predicts three (Asp31, His85, and His 110) while global conservation predicts none. ResBoost alone predicts Ser129 and Asn131 – residues which are known to be catalytic based on experimental evidence but are not listed in the CSA [26].

We analyzed the sensitivity of the predictions of the different methods on 1std for a fixed specificity. We chose a specificity of 90.2% corresponding to a value of *k *= 128. Thresholds for each of the other methods was chosen to achieve the same specificity. The predictions are shown in Figure 5. We see that ResBoost predicts all the residues listed in the CSA for 1std, i.e., Tyr30, Asp31, Tyr50, His85, and His110. Further, ResBoost also predicts Ser129 and Asn131 – residues which are not present in the CSA entry but have been experimentally validated [26]. ResBoost predicted these residues at *k *= 128 because of the second clause in ResBoost's logical expression: in a cluster and not hydrophobic and in a pocket with solvent accessible surface area > 35.36 Å^2^. Both ConSurf and ET correctly predict Asp31, His85, and His110 to be catalytic while incorrectly rejecting Tyr30. While ET correctly predicts Tyr50, ConSurf rejects Tyr50. Finally, global conservation fails to predict any of the catalytic residues. Interestingly, none of the other three methods predicts the residues Ser129 and Asn131.

**Figure 6:**
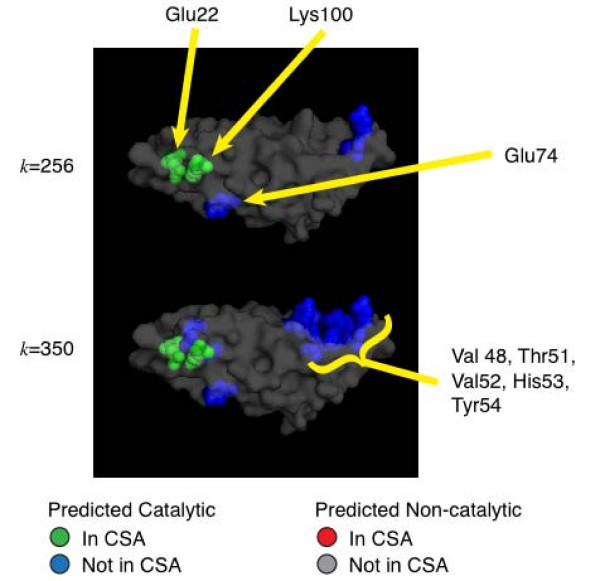
**ResBoost's catalytic residue predictions for dihydroneopterin aldolase from *Staphylococcus aureus ***(PDB ID: 2dhn). Results are shown for sensitivity/specificity trade-off parameter *k *= 256 and *k *= 350. ResBoost at *k *= 256 correctly predicts the known catalytic residues, Glu22 and Lys100, resulting in no false negatives. ResBoost also predicts Glu74, which though not listed as a catalytic residue in the CSA, exhibits a dramatic change in the affinities of the enzyme for the substrate or product analogues when mutated. At *k *= 350, ResBoost is more sensitive and detects residues located in a cleft away from the active site. Of these predicted residues, Tyr54 functions with Glu22 and Lys100 in organizing the catalytic center assembly while some of the other residues (Val48, Thr51, Val52, His53) are involved in electrostatic or hydrophobic interactions with the ligand in the crystal structure with neopterin (NP), an analog of 7,8-dihydroneopterin (DHNP) [28].

Our second example is an enzyme from *Staphylococcus aureus *– dihydroneopterin aldolase (PDB ID: 2dhn). Dihydroneopterin aldolase catalyzes the conversion of 7,8-dihydroneopterin (DHNP) to 6-hydroxymethyl-7,8-dihydropterin (HP) in the folate biosynthesis pathway. The folate biosynthesis pathway is present in bacteria, yeasts, and plants but is absent in mammals. This makes dihydropterin aldolase an ideal antimicrobial target [27]. Figure 6 shows the ResBoost predictions for two values of the trade-off parameter *k*, *k *= 256 (top) and *k *= 350 (bottom). ResBoost at *k *= 256 correctly predicts the known catalytic residues – Glu22 and Lys100. At *k *= 350, ResBoost predicts additional residues in a cleft located away from the active site. Of these predicted residues, Tyr54 functions with Glu22 and Lys100 in organizing the catalytic center assembly [28]. Other residues predicted in this cleft (Val48, Thr51, Val52, His53) are involved in electrostatic or hydrophobic interactions between neopterin (NP), an analog of DHNP, and its ligand [28]. A mutant of Glu74, another residue predicted by ResBoost, exhibits a dramatic change in the affinities of the enzyme for the substrate [29]. ResBoost classified this residue as catalytic because it is in a cluster and not hydrophobic and has a global conservation score > 0.9; ET and global conservation methods also predict this residue as catalytic.

We also investigated the robustness of ResBoost's predictions to protein conformational changes upon ligand binding. We ran ResBoost on the apo (catalytically inactive) and holo (catalytically active) forms of three enzymes, which were randomly selected from the data set. The first pair is glucosamine-6-phosphate deaminase (PDB ID: 1cd5) along with its holo form, binding ligands *N*-acetyl-*D*-glucosamine 6 phosphate and L(+) tartaric acid (PDB ID: 1fs5) [30]. The second pair is 3',5"-aminoglycoside phosphotransferase type IIIa (PDB ID: 1j7i) and its structure bound to substrates kanamycin A and ADP (PDB id 1l8t) [31]. The third pair is turkey egg lysozyme (PDB ID: 135l) and its holo form when bound to di-*N*-acetylchitobiose (PDB ID: 1lzy) [32]. These three enzymes have a combined total of 655 residues, of which 8 are labeled as catalytic in the Catalytic Site Atlas. For these known catalytic residues, ResBoost's predictions at *k *= 256 are identical for both the apo and holo forms with the exception of one residue: Lys44 of 3',5"-aminoglycoside phosphotransferase type IIIa. ResBoost correctly classifies this residue in the apo form but misclassifies it as non-catalytic for the holo form because the residue is not considered to be in a cluster in the holo form. At *k *= 350, ResBoost correctly classifies Lys44 as catalytic in both the apo and holo forms.

## Discussion

ResBoost characterizes catalytic residues using a compact logical expression based on an automatically selected subset of the provided rules of thumb. These rules provide insight into the dominant characteristics that differentiate catalytic and non-catalytic residues. ResBoost aims to encode intuition and observations into a computational framework; the results can then be used to narrow the list of candidate catalytic residues that can be validated using methods like mutagenesis experiments.

At all values of the sensitivity/specificity trade-off *k*, the dominant determinants of catalytic residues in ResBoost are evolutionary conservation scoring methods (clustering with ET and global conservation) and structure-based methods (clustering with ET and pocket area measurement). Residue hydrophobicity also played a role. Base classifiers that were not included from the ResBoost selection process include other evolutionary conservation scoring methods, solvent accessibility, and secondary structure. While these other classifiers are useful in isolation, they are highly correlated with other classifiers selected in earlier iterations in the ResBoost training phase and so do not appear in the first five base classifiers that form the ATrees.

We evaluated the performance of ResBoost on a randomly selected subset of 100 enzymes from the CSA. By randomly selecting our training set from a well-established independent database, we eliminate bias in the selection of the enzymes to validate our method. For 73% sensitivity, ResBoost has a False Positive Rate (FPR) of 5.7% while global conservation and Consurf have FPR's of 13% and ET has 8.9%. ResBoost cuts the FPR by 56% compared to global conservation and ConSurf and by 36% compared to ET. The implications of this difference are large for experimentalists attempting to narrow their list of mutagenesis targets. A FPR of 5.7% corresponds to 20 false positives on average per protein for our data set, and a FPR of 13% corresponds to 46 false positives per protein, a more than doubling of the number of mutagenesis experiments that must be considered.

The neural network methods of Gutteridge et al. [20] and Tang et al. [21] and the SVM approaches of Petrova and Wu [22], Youn et al. [23], and Pugalenthi et al. [24] also combine multiple predictors into a single classifier. However, their methods do not provide the ability to directly control the sensitivity vs. FPR trade-off. In one of the first methods to combine multiple predictors into a single classifier, Gutteridge et al. report a sensitivity of 56% at a precision of 14% [20]. Tang et al. reported a sensitivity to 73.2% using a genetic algorithm integrated neural network [21]. By using an SVM method, Youn et al. achieved a sensitivity of 65.3% at a precision of 14.4% on the same dataset as Gutteridge et al. and achieved 57.0% sensitivity at 18.5% precision for the ASTRAL 40 nonredundant dataset [23]. Petrova and Wu, using a SVM method to classify residues, reported results of 89% sensitivity for a false positive rate of 14% using cross-validation on a dataset composed of 79 structurally heterogeneous enzymes from the PIRSF protein families [22]. These results are based on a balanced dataset in which the majority of non-catalytic residues were discarded such that the number of catalytic residues equals the number of non-catalytic residues. Pugalenthi et al., using an SVM with a different set of features, achieved 88.6% sensitivity at a false positive rate of over 25% using 10-fold cross-validation [24]. While the methods like those of Youn et al., Pugalenthi et al., and Petrova and Wu can make predictions comparable to the results of ResBoost at fixed FPR's, they rely on a back-box rather than on logic expressions that intuitively characterize catalytic residues.

The CSA, a hand-curated dataset from primary literature, labels catalytic residues of a broad range of enzymes. The literature, however continues to accumulate evidence for additional residues not yet captured by CSA. The dihydroneopterin aldolase discussed in the previous section is a case in point, suggesting that the actual false positive rate of ResBoost may be lower than the false positive rate determined by comparison with CSA data.

The high false positive rates of all catalytic residue prediction methods are indicative of the challenge of characterizing and predicting catalytic residues solely from sequence and structural information. As new hypotheses to reduce the number of false positive predictions emerge, they should be integrated into catalytic residue prediction software. Because ResBoost relies on base classifiers, it provides an ideal platform to integrate these new hypotheses. Whereas adding new rules to other methods may degrade prediction accuracy [22], boosting is typically robust to the inclusion of new rules irrespective of their utility [33]. In future work, we plan to add base classifiers to ResBoost for additional rules of thumb based on residue flexibility, new clustering approaches, quaternary interactions, and THEMATICS [18]. Any base classifier has potential to improve the results of AdaBoost; it does not need to perform better than existing methods when executed independently. When integrated into ResBoost, the full potential of new rules of thumb can be harnessed.

## Conclusion

This paper presents ResBoost, a new computational method for identifying catalytic residues in enzymes. ResBoost uses AdaBoost and ATrees to learn the characteristics of catalytic residues and present them as an intuitive logical expression of simple rules. The logical expression combines rules of thumb for catalytic residue prediction that experimentalists typically use to manually narrow the list of candidate residues, including residue evolutionary conservation, 3D clustering, solvent accessibility, pocket accessibility, and hydrophilicity. The method also provides precise control over the inherent trade-off between sensitivity and specificity. We evaluated ResBoost using cross-validation on a dataset of 100 enzymes from the hand-curated Catalytic Site Atlas. We also illustrated the ability of ResBoost to identify recently validated catalytic residues not listed in the CSA. By combining multiple rules in a single unifying framework, ResBoost predicts catalytic residues with greater accuracy than is possible using individual rules in isolation.

## Methods

### Base classifiers considered during training

We describe the base classifiers used in our method in the subsections below. The base classifiers roughly fall into three categories based on the primary source of information they use to classify residues:

• Evolutionary conservation scoring: Classify the residues based on the patterns of sequence evolution. Base classifiers in this category include Global Conservation, Evolutionary Trace, and ConSurf.

• Structural information: Classify residues using various features derived from the structure of the protein. Base classifiers in this category include single-linkage clustering, solvent accessibility, pocket accessibility, and secondary structure.

• Residue-based information: Classify residues using properties of individual amino acids. Base classifiers in this category include catalytic propensity, residue charge, and residue hydrophilicity.

ResBoost considered two types of base classifiers: threshold classifiers and binary classifiers. A threshold classifier first computes a value *v*_*i *_for each residue *x*_*i*_, such as an Evolutionary Trace score or a solvent accessible area. Given a threshold *p*, for each residue *x*_*i *_the threshold classifier returns classification TRUE if *v*_*i *_≥ *p *and FALSE otherwise. A binary classifier only tests whether each residue passes a particular test and returns TRUE if it does and FALSE otherwise. Both of these base classifiers are a type of decision stump [34]. For threshold classifiers, the threshold is a variable that is optimized at each iteration of the training phase of the AdaBoost algorithm.

The predictions of several of these base classifiers are highly correlated. For example, solvent accessibility and presence in a pocket are highly correlated; residues must have non-zero solvent accessibility in order to be in a pocket. Also, the residue-based classifier for catalytic propensity is highly correlated with residue charge and hydrophilicity. The boosting method described later in this section selects and combines a low-correlation subset of these base classifiers to predict catalytic residues with high accuracy.

Below we provide details on the base classifier types that were included in the final ResBoost classifiers for *k *= 256, 128, or 64. Details on the additional base classifier types that were considered during the boosting algorithm but not selected are described in the online supporting information [see Additional file 1]. A total of 2722 base classifiers were considered across the categories described above.

#### Global conservation

Catalytic residues are generally more conserved across homologues than non-catalytic residues [35]. To compute conservation scores for the residues in an enzyme, we constructed a multiple sequence alignment for the enzyme and homologues and defined the global conservation for a particular residue as the percent of sequences in the alignment for which the amino acid at the residue's position is identical to the amino acid in the enzyme.

We constructed the multiple sequence alignment for each enzyme in our dataset by gathering homologues using PSI-BLAST [36] against the UniProt database [37]. PSI-BLAST was run for 4 iterations with an E-value cutoff of 10^-4^. In cases where PSI-BLAST returned a large number of homologues, the top 1000 hits were retained. The homologues were aligned to the query using MUSCLE [38]. Columns in the alignment corresponding to gaps in the query were removed. Identical sequences were removed. For consistency across enzymes, we normalized global conservation scores for each enzyme separately so the highest scoring residue is given a score of 1 and the lowest scoring entry is 0.

#### Evolutionary trace

Evolutionary Trace (ET), first introduced by Lichtarge et al. in 1996 [6], ranks the relative functional importance of residues in a protein sequence using evolutionary tree analysis. Recent improvements combine evolutionary and entropic information from multiple sequence alignments to create a hybrid scoring method that improves residue scores relative to the original method [8]. Yao et al. demonstrated that the location of residues with high Evolutionary Trace scores is correlated with the location of functional sites [7].

We obtained ET scores for the residues of each enzyme in our dataset using the Evolutionary Trace Report Maker available online at the Baylor College of Medicine [8]. We provided the PDB ID of each enzyme and no additional information. For consistency with other base classifiers which consider higher scores as better, we take the negative of each ET score provided by the report maker. For consistency across enzymes, we normalize the negated scores so the highest scoring entry is 1 and the lowest scoring entry is 0 for each enzyme.

#### Single-linkage clustering

Past studies indicate that catalytic residues are spatially clustered in a protein's tertiary structure [3]. We combine evolutionary conservation scoring with spatial clustering to help classify residues as catalytic.

Single-linkage clustering is a type of nearest-neighbor clustering [39]. We consider each residue as a node in a graph. For each node, we construct an edge to all other nodes that are spatially closer than some distance threshold. Two nodes *r*_1 _and *r*_2 _are considered connected if it is possible to start at *r*_1 _and traverse edges in the graph to reach node *r*_2_. A set of nodes for which all pairs are connected forms a cluster. A key advantage of this clustering algorithm compared to centroid-based clustering is that it can identify clusters that are not spherically symmetric.

Our single-linkage clustering implementation requires five parameters: *C*, *S*, *D*, *R*, and *M*. Parameter *C *specifies an evolutionary conservation score type (Global Conservation, Evolutionary Trace, or ConSurf). As a filtering step, if the evolutionary score *s*_*i *_for residue *x*_*i *_is less than *S *then we remove residue *x*_*i *_from consideration for clustering. The single-linkage clustering algorithm clusters the remaining residues using *D *as the distance threshold. We define the distance between two residues as the shortest Euclidean distance between any atom of the first residue and any atom of the second residue. Atom coordinates were obtained from PDB files downloaded from the Protein Data Bank [40]. After generating the clusters, we remove small clusters containing fewer than *R *residues. We then sort the list of clusters based on a cluster score, which equals the sum of the evolutionary conservation scores for each residue in the cluster. If the number of clusters is greater than *M*, we remove low-scoring clusters so that only *M *clusters remain.

Given the evolutionary conservation score type *C*, the minimum evolutionary conservation score threshold *S*, the distance threshold *D*, the minimum number of residues/cluster *R*, and the maximum number of clusters *M*, the single-linkage clustering binary classifier computes the clusters and returns TRUE if a residue is in a cluster and FALSE otherwise. We generate many such binary classifiers for different values of the parameters. Specifically, we consider 2700 such binary classifiers for 3 evolutionary conservation score types *C*, 15 values of *S *in the range of 0.7 to 1.0, 5 values of *D *in the range of 3Å to 4Å, *R *= 1, 2, or 3 residues, and *M *= 1, 2, 3, or 4 clusters.

For the logical expressions presented in the Results section, the parameters selected for each value of *k *by the AdaBoost algorithm were: evolutionary score type *C *= Evolutionary Trace, minimum score *S *= 0.73, distance threshold *D *= 3.25 Å, minimum number of residues *R *= 2, and maximum number of clusters *M *= 5.

#### Presence in a pocket

Pockets, also known as clefts or cavities, are concave regions on a protein's surface [41]. The shape of the molecular surface of a protein has long been known to influence the protein's interaction with other molecules, including water molecules, ligands, DNA, and other proteins [42-44]. In particular, ligands often bind inside protein pockets [45,46]. Using a sample of 178 enzymes in the CSA, Bartlett et al. showed that 93% of enzymes had at least one catalytic residue inside a cleft [35].

Several algorithms have been developed to identify and visualize protein pockets [46,47]. We use the CASTp algorithm, which measures protein pocket sizes analytically using precise computational geometry methods, including alpha shape and discrete flow theory [46]. The method both identifies the atoms forming pockets and computes the interior volume and molecular surface area of the pockets. We used the CASTp server available online to obtain pocket information for proteins in our dataset [41]. For each pocket, CASTp provides four measurements: solvent accessible surface area, molecular surface area, solvent accessible volume, and molecular volume. CASTp also identifies all residues in contact with each pocket.

We create a threshold classifier for each of the four measurement types obtained from CASTp. Given a threshold *V*, the threshold classifier classifies a residue as TRUE if the residue is in contact with a pocket that has a measurement value (volume or surface area) greater than or equal to *V *and FALSE otherwise. If a residue is not in contact with a CASTp-identified pocket, we consider it to be attached to a pocket of zero area and volume. If a residue is in multiple pockets (some CASTp pockets are superset of other pockets), we consider it to be in contact with the pocket with largest area or volume (depending on the measurement type used by the classifier).

#### Residue hydrophobicity

As in Bartlett et al. [35], we classified residues of type A, F, G, I, L, M, P, and V as hydrophobic. Bartlett et al. showed that charged residues are more likely to be catalytic than hydrophobic residues. We defined a base classifier for hydrophobicity that classifies a residue as TRUE if the residue is charged and FALSE otherwise.

### Training phase

**Figure 7:**
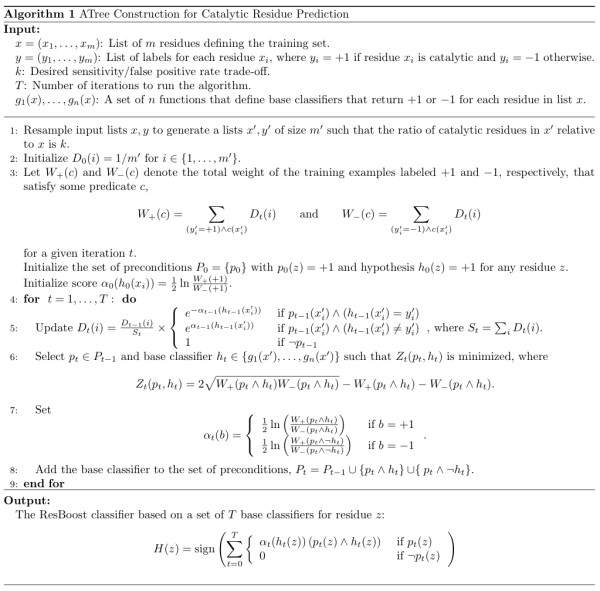
**The alternating decision tree learning algorithm for oversampled catalytic residue prediction and the resulting ResBoost classifier**.

We define our implementation of AdaBoost-based Alternating Decisions Trees (ATrees) for catalytic residue prediction in Figure 7. The input to the method is a list *x *= (*x*_1_, ..., *x*_*m*_) containing all the residues from each protein in the training set. The training set, which we fully describe below, is based on a random sample of hand-curated proteins from the CSA. We assign a label *y*_*i *_to each residue *x*_*i *_in the training set, where *y*_*i *_= +1 if residue *x*_*i *_is labeled as a catalytic residue in the CSA and *y*_*i *_= -1 otherwise.

Throughout the paper, we assume TRUE is equivalent to +1 and FALSE is equivalent to -1. The method also requires an input parameter *k *that specifies a desired trade-off between sensitivity and specificity, a parameter *T *that sets the number of training iterations to execute, and a list of *n *base classifiers *g*_1_(*x*), ..., *g*_*n*_(*x*). Given a list of residues *x*, each base classifier, which we defined above, returns a predicted classification (+1 or -1) for each residue.

The first step of the method is to resample the training set based on input parameter *k*. The AdaBoost and ATree algorithms, as originally presented [34,48], consider each data input with equal importance when minimizing classification errors, which would inherently result in a far higher number of false positives than false negatives for catalytic residue predictions due to the fact that most residues in enzymes are not catalytic. To address this issue, we use oversampling to provide control over the ratio of catalytic residues to non-catalytic residues during training. Given an integer parameter *k *≥ 1, we duplicate catalytic residues in the training set *k *times to generate residue list *x' *of size *m' *and corresponding labels *y'*. The result is that parameter *k *provides precise control over the trade-off of sensitivity to the number of false positives, as illustrated in the Results section.

The next step is to generate the ResBoost classifier by providing the oversampled training set to the ATree learning algorithm. We utilize the ATree implementation in JBoost, a machine learning program implemented in the Java programming language that is available freely online [49]. The ATree algorithm was originally devised by Freund and Mason [34] and is based on confidence-rated AdaBoost [50,51]. The underlying principle of the learning algorithm is to iteratively combine the base classifiers. The only parameter of the algorithm is *T*, the number of base classifiers to combine. Once this parameter is specified, the algorithm runs for *T *iterations over the base classifiers, adding one base classifier at each iteration.

The ATree maintains a set of hypotheses, where each hypothesis is the conjunction of a precondition and a base classifier. A hypothesis can be written as *p *∧ *h*, where ∧ (logical AND) denotes the conjunction of a precondition *p *and a base classifier *h*. Each node in the tree corresponds to a base classifier or the negation of a base classifier. The precondition of a node in the tree is the conjunction of the base classifiers (or the negation of the base classifiers) along the path from the node to the root.

To initialize the method, we define a root node with a hypothesis of TRUE, meaning that all examples satisfy the root's precondition. At each iteration, a new base classifier is added. The path leading from the root to any node in the tree (including the zero length path to the root) can serve as a precondition. For instance, if the tree already has a node with the hypothesis "is hydrophobic" then we can use this hypothesis as a precondition and conjunctively add a new base classifier to it that tests conservation, resulting in two new hypotheses, "is hydrophobic and is conserved" and "is hydrophobic and is not conserved." These two new hypotheses are added as nodes under the node "is hydrophobic". All the base classifiers are tested on all leaves in the tree (i.e. all available preconditions) and the base classifier that best discriminates the catalytic residues from the non-catalytic residues is added to the tree. The formal criteria for measuring discrimination, provided by *Z*_*t *_in Figure 7, was shown to be optimal for this type of classification [51]. Importantly, this discrimination is done with respect to the weights of the examples *D*_*t*_(*i*); if an example has been classified incorrectly at previous iterations, its weight will be larger than the weight of examples that were previously correctly classified. This makes it more important for each new hypothesis to correctly classify residues that were misclassified in previous iterations. Furthermore, each hypothesis is given a prediction-dependent score *α*_*t*_, as shown in Figure 7, where a higher score corresponds to higher confidence in the prediction.

The major difference between ATrees and standard boosting and machine learning algorithms is that hypotheses may be combined into conjunctive clauses (logical AND operations). This makes ATrees significantly more powerful, but also more prone to overfitting the data. To show that we do not overfit our dataset, we perform standard cross-validation experiments which we discuss in the Results section.

The output of the training phase is the ResBoost classifier, a weighted logical expression of *T *base classifiers. The classifier *H*(*z*) consists of the output of *T *hypotheses, each weighted by their prediction-dependent confidence score *α*_*t *_determined from the training phase. Using this output, any new protein residue *z *can be classified by computing *H*(*z*) as defined in Figure 7.

We can simplify the ResBoost classifier *H*(*z*) in Figure 7 to write an equivalent but more compact logical expression of the rules of thumb. Since *H*(*z*) is based on *T *base classifier evaluations and each evaluation can be TRUE or FALSE, we can write the logical expressions for all 2^*T *^possible outcomes. For each outcome that yields a prediction of *H*(*z*) > 0, we simplify the logic using DeMorgan's laws of logical equivalences [25]. As illustrated in the Results section, this results in a compact, intuitive set of necessary conditions for a residue to be predicted as catalytic.

We executed the training phase for *k *= 1, 2, 4, 8, 16, 32, 64, 128, 150, 175, 200, 225, 256, 350, 512 and 1024. We set the number of iterations *T *= 5 for all values of *k*, although further research is required to determine an optimal *T*.

The learning algorithm required approximately one hour for each fold of the cross validation experiment. Applying our method to new proteins requires approximately two seconds per protein when all conservation data is provided by the user.

### Dataset

To train and evaluate the method, we created a dataset of 100 enzymes for which a label (catalytic or non-catalytic) is available in the literature for each residue. We used a subset of the Catalytic Site Atlas for this dataset. As of 2004, the CSA contained 177 hand-annotated entries and 2608 homologous entries which cover approximately 30% of all EC numbers found in the PDB [4]. We only considered enzymes in the CSA for which catalytic residues had been identified experimentally and published in the primary literature. We then randomly selected 100 sequence-divergent enzymes for which we could obtain PDB files, DSSP files, ET scores, ConSurf scores, and CASTp pocket information files. We defined two enzymes to be sequence divergent if the BLAST E-value [36] for the pair was greater than 1.0 when run against the PDB database, which contained 28,876 sequences at the time of testing. For enzymes composed of multiple chains, we only considered a single randomly selected chain for which ET and ConSurf scores were available. The final dataset included 36,278 total residues, of which 316 were labeled as catalytic in the CSA.

Details on the enzymes in the dataset are provided in the online supporting information [see ResBoostDataset.pdf]. In summary, the PDB ID's for the proteins (and their included chain) are: 1qdl-A, 1b8g-B, 1fps, 1mfp-A, 1arz-A, 1d3g-A, 1qb4-A, 1kas, 1a7u-A, 1dii-A, 1grc-A, 1uag, 1zio, 1ay4-A, 1bou-B, 1b73-A, 1q91-A, 1daa-A, 1cd5-A, 1aq0-A, 1fdy-A, 1ecx-A, 1qba, 1kc7-A, 1pja-A, 1hdh-A, 1geq-B, 1exp, 1aql-A, 1b66-A, 1qum-A, 1d7r-A, 1qd6-C, 1cz1-A, 5enl, 1ah7, 1pmi, 1og1-A, 1tph-1, 1ey2-A, 1ahj-A, 1pnl-B, 1ir3-A, 1oe8-B, 1d0s-A, 1gim, 1std, 1a0i, 1aop, 1qcn-B, 1get-B, 1uqt-A, 1m6k-A, 1trk-A, 1sme-A, 2ts1, 1ps9-A, 1ecl, 1ef0-A, 1aj0, 2dhn, 1akd, 7odc-A, 135l, 1fcb-A, 1cbg, 1f8m-A, 1aj8-A, 1cel-A, 1l8t-A, 1jms-A, 1mrq-A, 1qh9-A, 1mlv-B, 1mhl-D, 1f75-A, 1d6o-A, 1b57-A, 1chd, 1bwz-A, 1nln-A, 1nba-A, 1cmx-A, 1xik-B, 1lnh, 1opm-A, 1lxa, 1a50-B, 1yve-L, 1d2r-A, 1bt1-A, 1ab8-A, 1dbt-A, 1amo-A, 1p4r-A, 1eyp-A, 1do8-A, 1oyg-A, 1rbn, and 1qmh-B.

### Evaluation

We evaluate the predictions of ResBoost by considering the labels from the CSA as ground truth. A prediction is a true positive (TP) if and only if the predicted classification is TRUE and the residue is labeled catalytic in the CSA. A prediction is a false negative (FN) if and only if the predicted classification is FALSE but the residue is labeled catalytic in the CSA. A prediction is a false positive (FP) if the predicted classification is TRUE but the residue is labeled FALSE as determined from the CSA. A prediction is a true negative (TN) if the predicted classification is FALSE and the residue is labeled FALSE as determined from the CSA.

We evaluate the results using the measures of sensitivity, specificity, FPR, precision, and recall. Sensitivity is defined by:



Specificity is defined by:



The false positive rate (FPR) is defined by:



Ideally, the sensitivity would equal 1 while the FPR would equal 0. But for most problems, the FPR increases as the desired sensitivity is increased. Precision is the fraction of the predicted catalytic residues that are true catalytic residues:



Recall is identical to sensitivity: the fraction of the true catalytic residues that are successfully predicted as catalytic by the method. Ideally, both the precision and recall equal 1.

We performed 10-fold cross validation (CV) to verify the robustness of our method. Traditional 10-fold CV partitions the dataset into 10 equally sized sets and uses 9 of these sets to learn the classifier and 1 to examine test error. Averaging test-error across the 10 learned classifiers and 10 test sets provides an estimate of the robustness of the classification method. In addition, this provides a heuristic for selecting the appropriate number of iterations *T *[34].

Because we evaluated the method using 10-fold cross validation, each training set included on average 9/10 of the residues and each evaluation dataset included 1/10 of the residues. This means the training sets averaged 32,650 total residues with 284 catalytic residues, and the evaluation sets averaged 3,628 total residues with 32 catalytic resides.

For CV results to be generalizable to new unseen data, it is necessary that the set used for learning and the set used for testing be independent for each fold. To ensure that this requirement is satisfied, we partitioned the dataset according to proteins, not according to residues. Thus, we avoided splitting neighboring catalytic residues that may share similar properties across the learning set and test set. In addition, we did not oversample or balance the test sets.

For comparison, we also classified residues in our dataset using global conservation and the publicly available Evolutionary Trace and ConSurf methods. These methods generate scores for each residue, but they do not explicitly classify each residue as catalytic or non-catalytic. As described for the base classifiers, we obtained scores for each method and normalized all scores for each score type on a protein by protein basis rather than considering residue ranks. To compare predictions across methods in a standard manner, we apply a score threshold and assign all residues with score above the threshold a classification of TRUE and all other residues a classification of FALSE. We can vary the score threshold to produce a trade-off between sensitivity and specificity for each method. By using score thresholding, we focus solely on the automatic residue prediction capabilities of global conservation, ET, and ConSurf; we do not interpret or analyze other information, such as evolutionary rates or catalytic surfaces patches, that these methods provide.

## Authors' contributions

RA and KS conceived the original method and designed the experiments. RA and AA carried out the software development, performed experiments, and made modifications to the original algorithm. YF and AA contributed expertise in the AdaBoost and Alternating Decision Trees algorithms. SS and CD assisted in data analysis. RA, AA, SS and KS analyzed the data and wrote the paper.

## Supplementary Material

Additional file 1 **ResBoost dataset**. Details on the enzymes in the ResBoost dataset and additional base classifiers. Click here for file
