# Supplementary material for: ResBoost: characterizing and predicting catalytic residues in enzymes
Source: BMC Bioinformatics. 2009 Jun 27;10:197. doi: 10.1186/1471-2105-10-197 (PMC2713229; doi:10.1186/1471-2105-10-197)
Supplement: Additional file 1 — ResBoost dataset. Details on the enzymes in the ResBoost dataset and additional base classifiers. [file 1471-2105-10-197-S1.pdf]

Table S1: ResBoost Dataset.

PDB ID	Chain	Molecule	EC number	Sequence length	Catalytic Residues
135l		Turkey egg white lysozyme	3.2.1.17	129	Glu35, Asp52
1a0i		DNA ligase	6.5.1.1	348	Lys34
1a50	B	Tryptophan synthase (beta chain)	4.2.1.20	396	Asp305, Lys167, His86, Lys87
1a7u	A	Chloroperoxidase t	1.11.1.10	277	Met99, His257, Phe32, Asp228, Ser98
1ab8	A	Adenylyl cyclase	4.6.1.1	220	Arg1029
1ah7		Phospholipase c	3.1.4.3	245	Asp55
1ahj	A	Nitrile hydratase (subunit alpha)	4.2.1.84	207	Cys113, Ser114, Cys115
1aj0		Dihydropteroate synthase	2.5.1.15	282	Arg255, Asn22, Arg63
1aj8	A	Citrate synthase	4.1.3.7	371	His262, Asp312, His223
1akd		Cytochrome p450cam	1.14.15.1	414	Asp251, Thr252
1amo	A	NADPH-cytochrome p450 reductase	1.6.2.4	615	Asp675, Ser457, Cys630
1aop		Sulfite reductase hemoprotein	1.8.1.2	497	Fs4575, Arg83, Lys215, Srm580, Cys483, Lys217, Arg153
1aq0	A	1,3-1,4-beta-glucanase	3.2.1.73	306	Glu280, Glu232, Glu288, Lys283
1aql	A	Bile-salt activated lipase	3.1.1.13	532	Ala108, Asp320, His435, Ser194, Ala195, Gly107
1arz	A	Dihydrodipicolinate reductase	1.3.1.26	273	His159, Lys163
1ay4	A	Aromatic amino acid aminotransferase	2.6.1.57	394	Trp140, Asp222, Lys258
1b57	A	Fructose-bisphosphate aldolase II	4.1.2.13	358	Asp109, Zn360, Asn286, Glu182
1b66	A	6-pyruvoyl tetrahydropterin synthase	4.6.1.10	140	Glu133, Cys42, Glu133, Cys42, Asp88, His89
1b73	A	Glutamate racemase	5.1.1.3	254	Cys70, Asp7, Ser8, Cys178
1b8g	B	1-aminocyclopropane-1-carboxylate synthase	4.4.1.14	429	Asp230, Lys273, Tyr145
1bou	B	4,5-dioxygenase beta chain	1.13.11.8	302	His195
1bt1	A	Catechol oxidase	1.10.3.1	345	Glu236
1bwz	A	Diaminopimelate epimerase	5.1.1.7	274	Glu208, His159, Cys217, Cys73
1cbg		Cyanogenic beta-glucosidase	3.2.1.21	490	Glu397, Glu183, Asn324
1cd5	A	Glucosamine 6-phosphate deaminase	5.3.1.10	266	Glu148, Asp141, His143, Asp72
1cel	A	1,4-beta-d-glucan cellobiohydrolase i	3.2.1.91	434	Asp214, Glu217, His228, Glu212
1chd		Cheb methylesterase	3.1.1.61	203	Ser164, Thr165, Asp286, His190, Met283
1cmx	A	Ubiquitin yuh1-ubal	3.1.2.15	236	Asp181, Gln84, His166, Cys90
1cz1	A	Exo-b-(1,3)-glucanase	3.2.1.58	394	Glu192, Glu292
1d0s	A	Nicotinate mononucleotide:5,6-dimethylbenzimidazole phosphoribosyltransferase	2.4.2.21	356	Glu317

1d2r	A	Tryptophanyl tRNA synthetase	6.1.1.2	326	Lys192, Lys195
1d3g	A	Dihydroorotate dehydrogenase	1.3.3.1	367	Phe149, Lys255, Ser215, Thr218
1d6o	A	Fk506-binding protein	5.2.1.8	107	Asp37, Ile56, Tyr82
1d7r	A	2,2-dialkylglycine decarboxylase (pyruvate)	4.1.1.64	433	Lys272, Asp243, Trp138
1daa	A	D-amino acid aminotransferase	2.6.1.21	282	Leu201, Glu177, Lys145
1dbt	A	Orotidine 5'-phosphate decarboxylase	4.1.1.23	239	Asp60, Lys62
1dii	A	P-cresol methylhydroxylase	1.17.99.1	521	Tyr473, Arg474, Glu427, Tyr95, His436, Glu380
1do8	A	Malic enzyme	1.1.1.39	564	Asp278, Lys183, Tyr112
1ecl		Escherichia coli topoisomerase i	5.99.1.2	597	Tyr319, Asp111, Glu9, His365
1ecx	A	Aminotransferase	No	384	His99, Asp177, Lys203
1ef0	A	PI-Scei endonuclease	3.6.1.34	462	Cys455, Ala1, Asn76, Gly433, Thr78, Ile434, His79, Ala454
1exp		Beta-1,4-d-glycanase cex-cd	3.2.1.91,	312	His205, Glu127, Glu233, Asp235
1ey2	A	Homogentisate 1,2-dioxygenase	1.13.11.5	471	His365, His292
1eyp	A	Chalcone-flavonone isomerase 1	5.5.1.6	222	Thr190, Asn113, Thr48, Tyr106
1f75	A	Undecaprenyl pyrophosphate synthetase	2.5.1.31	249	Arg197, Arg203, Arg33, Arg42
1f8m	A	Isocitrate lyase	4.1.3.1	429	His180, Cys191, Arg228
1fcb	A	Flavocytochrome b2	1.1.2.3	511	Tyr254, Arg376, Tyr143, Asp282, His373
1fdy	A	N-acetylneuraminate lyase	4.1.3.3	297	Lys165, Ser47, Thr48
1fps		Farnesyl diphosphate synthase	2.5.1.10	348	Phe253, Arg126
1geq	B	Tryptophan synthase alpha-subunit	EC	248	Glu36, Asp47, Tyr161
1get	B	Glutathione reductase	1.6.4.2	450	His439, Glu444, Glu181, Cys47, Tyr177, Lys50, Cys42
1gim		Adenylosuccinate synthetase	6.3.4.4	431	Asp13, Gln224, His41
1grc	A	Glycinamide ribonucleotide transformylase	2.1.2.2	212	His108, Ser135, Asp144, Asn106
1hdh	A	Arylsulfatase	3.1.6.1	536	Fgl51, Lys375, Arg55, Ca1528, Lys113, His211, Asp317, His115
1ir3	A	Insulin receptor	2.7.1.112	306	Arg1136, Asp1132
1jms	A	Terminal deoxynucleotidyltransferase	2.7.7.31	381	Mg701, Asp434
1kas		Beta-ketoacyl acp synthase II	2.3.1.41	412	Phe400, His303, His340, Cys163
1kc7	A	Pyruvate phosphate dikinase	2.7.9.1	873	His455, Cys831
1l8t	A	Aminoglycoside 3'-phosphotransferase	2.7.1.95	263	Lys44, Asp190
1lnh		Lipoxygenase-3	1.13.11.12	857	Asn713
1lxa		UDP n-acetylglucosamine o-acyltransferase	2.3.1.129	262	His125
1m6k	A	Beta-lactamase oxa-1	3.5.2.6	251	Ser67, Kcx70

1mfp	A	Enoyl-[acyl-carrier-protein] reductase [nadh]	1.3.1.9	262	Tyr156, Lys163
1mhl	D	Myeloperoxidase	1.11.1.7	466	Arg239
1mlv	B	Ribulose-1,5 biphosphate carboxylase/oxygenase large subunit n-methyltransferase	2.1.1.127	444	Tyr287
1mrq	A	Aldo-keto reductase family 1 member c1	1.1.1.149	323	His117, Lys84, Tyr55, Asp50
1nba	A	N-carbamoylsarcosine amidohydrolase	3.5.1.59	264	Ala172, Asp51, Thr173, Lys144, Cys177
1nln	A	Adenain	3.4.22.39	204	His54, Glu71, Cys122, Gln115
1oe8	B	Glutathione s-transferase	2.5.1.18	211	Tyr10
1og1	A	T-cell ecto-ADP-ribosyltransferase 2	2.4.2.31	226	Glu189, Glu159, Arg184, Ser147
1opm	A	Peptidylglycine alpha-hydroxylating monooxygenase	1.14.17.3	310	His108, His242, Gln170
1oyg	A	Levansucrase	2.4.1.10	447	Glu342, Asp247, Asp86
1p4r	A	Bifunctional purine biosynthesis protein PURH	2.1.2.3,	592	Asn431, His592, Lys266, His267, Ile126, Gly127, Tyr104, Lys137, Lys66
1pja	A	Palmitoyl-protein thioesterase 2 precursor	3.1.2.22	302	Leu45, Ser111, Gln112, His283, Asp228
1pmi		Phosphomannose isomerase	5.3.1.8	440	Glu294, Arg304, Gln111
1pnl	B	Penicillin amidohydrolase	3.5.1.11	557	Ala69, Asn241, Ser1
1ps9	A	2,4-dienoyl-coa reductase	1.3.1.34	671	Tyr166, His252
1q91	A	5(3)-deoxyribonucleotidase	3.1.3.5	197	Asp43, Asp41
1qb4	A	Phosphoenolpyruvate carboxylase	4.1.1.31	883	Arg581, Arg713, Arg396
1qba		Chitobiase	3.2.1.52	858	Glu540, Asp539
1qcn	B	Fumarylacetoacetate hydrolase	3.7.1.2	421	Arg737, His633, Gln740, Glu864, Lys753
1qd6	C	Outer membrane phospholipase (ompla)	3.1.1.32	240	His142, Ser144, Gly146
1qdl	A	Anthranilate synthase (TrpE-subunit)	4.1.3.27	422	His306, His306
1qh9	A	2-haloacid dehalogenase	3.8.1.2	232	Asp180, Ser118, Arg41, Asp10
1qmh	B	RNA 3'-terminal phosphate cyclase	6.5.1.4	347	His309
1qum	A	Endonuclease iv	3.1.21.2	285	Glu261
1rbn		Ribonuclease a	3.1.27.5	124	His12, His119, Phe120, Lys41
1sme	A	Plasmepsin II	3.4.23.39	329	Asp214, Ser37, Thr217, Asp34
1std		Scytalone dehydratase	4.2.1.94	172	His110, His85, Tyr30, Asp31, Tyr50
1tph	1	Triosephosphate isomerase	5.3.1.1	247	Asn11, Glu165, Lys13, His95, Gly171
1trk	A	Transketolase	2.2.1.1	680	His263, His30
1uag		UDP-n-acetylmuramoyl-l-alanine/:d-glutamate ligase	6.3.2.9	437	His183, Asn138, Lys115

1uqt	A	Alpha,alpha-trehalose-phosphate synthase	2.4.1.15	482	Asp361, His154
1xik	B	Protein r2 of ribonucleotide reductase	1.17.4.1	375	Tyr122
1yve	L	Acetohydroxy acid isomeroreductase	1.1.1.86	524	Glu496
1zio		Adenylate kinase	2.7.4.3	217	Lys13, Arg127, Arg160, Asp162, Arg171, Asp163
2dhn		7,8-dihydroneopterin aldolase	4.1.2.25	121	Lys100, Glu22
2ts1		Tyrosyl-tRNA synthetase	6.1.1.1	419	Lys230, Arg86, Lys233, Lys82
5enl		Enolase	4.2.1.11	436	Lys345, Glu168, Glu211, His373
7odc	A	Ornithine decarboxylase	4.1.1.17	424	His197, Lys69, Glu274

ResBoost additional base classifiers

ConSurf.

ConSurf, like ET, uses ideas from evolution to identify residues of functional importance. Based on the Rate4Site tool, ConSurf estimates the rate of evolution of each residue of the protein from the sequence and phylogenetic information, and then maps these rates onto the molecular surface of the protein to help identify patches that may be functionally important [1, 2].

We obtained ConSurf scores from version 3.0 [2] by specifying the PDB ID and chain and using all the default settings (including ConSurf's pre-computed multiple sequence alignments based on MUSCLE [3] and trees based on the neighbor joining algorithm [4]). If the protein chains had less than the required number of 5 unique PSI-BLAST hits, we changed the default ConSurf settings to use UniProt instead of the standard Swiss-Prot (this was required for only 3 of the 100 enzymes in our dataset). For consistency across enzymes, we normalized ConSurf scores so the highest scoring residue is 1 and the lowest scoring entry is 0 for each enzyme.

Solvent accessibility.

Catalytic residues must be at least somewhat solvent accessible in order to perform their biochemical function. We obtain solvent accessibility scores using DSSP [5], which is available from the PDB [6]. DSSP provides the surface area a_i that is in contact with the solvent for each residue x_i. Given a threshold A, the solvent accessibility threshold classifier classifies a residue i as TRUE if $a_i \geq A$ and FALSE otherwise.

The lack of solvent accessibility as measured by DSSP does not imply that a residue cannot be catalytic. Due to the complexity of enzyme interactions and the limitations of DSSP, some residues that are labeled as not solvent accessible in a static solved protein structure may in fact contact atoms in the solvent. This was shown to be the case for some catalytic residues in the CSA [7].

Secondary structure.

Catalytic residues have been observed in all secondary structures of enzymes. However, the proportion of residues that are catalytic in these secondary structures is not the same across all secondary structures. In particular, residues on alpha helices are somewhat less likely to be catalytic than residues on turns, loops, and coils [7]. Using DSSP [5], we classified each residue as being in an alpha helix, a beta sheet, or coil/other. We then defined one base classifier for each secondary structure type that classifies a residue as TRUE if the residue is in that secondary structure and FALSE otherwise.

Catalytic propensity.

Bartlett et al. measured the frequency of each amino acid type for all protein residues in the CSA and compared this with the frequency of each amino acid type among the catalytic residues in the database [7]. The frequencies provided quantitative support for an intuition that many biologists already had: nonpolar amino acids such as alanine and valine are rarely catalytic while polar amino acids such as histidine and glutamine are often catalytic.

We considered two types of catalytic propensity, side-chain and main-chain. For each type, we built a table of catalytic propensities [7] and assigned each residue x_i a catalytic propensity value c_i based on its amino acid. Given a threshold C, each catalytic propensity threshold classifier classifies a residue x_i as TRUE if $c_i \geq C$ and FALSE otherwise.

Residue charge.

As in Bartlett et al. [7], we classified residues of type H, R, K, E, and D as charged. We defined a base classifier for charge that classifies a residue as TRUE if the residue is charged and FALSE otherwise.

Residue polarity.

As in Bartlett et al. [7], we classified residues of type Q, T, S, N, C, Y, and W as polar. We defined a base classifier for polarity that classifies a residue as TRUE if the residue is polar and FALSE otherwise.

References

[1] Glaser F, Pupko T, Paz I, Bell RE, Bechor-Shental D, Martz E, Ben-Tal N: **ConSurf: identification of functional regions in proteins by surface-mapping of phylogenetic information**. *Bioinformatics* 2003, **19**:163–164.

[2] Landau M, Mayrose I, Rosenberg Y, Glaser F, Martz E, Pupko T, Ben-Tal N: **ConSurf 2005: the projection of evolutionary conservation scores of residues on protein structures**. *Nucleic Acids Res.* 2005, **33**:W299–W302.

[3] Edgar RC: **MUSCLE: multiple sequence alignment with high accuracy and high throughput**. *Nucleic Acids Research* 2004, **32**(5):1792–1797.

[4] Saitou N, Nei M: **The neighbor-joining method: a new method for reconstructing phylogenetic trees**. *Molecular Biology and Evolution* 1987, **4**(4):406–425.

[5] Kabsch W, Sander C: **Dictionary of Protein Secondary Structure: Pattern Recognition of Hydrogen-Bonded and Geometrical Features**. *Biopolymers* 1983, **22**:2577–2637.

[6] Berman H, Westbrook J, Feng Z, Gilliland G, Bhat T, Weissig H, Shindyalov I, Bourne P: **The Protein Data Bank**. *Nucleic Acids Res.* 2000, **28**:235–242.

[7] Bartlett GJ, Porter CT, Borkakoti N, Thornton JM: **Analysis of catalytic residues in enzyme active sites**. *J. Mol. Biol.* 2002, **324**:105–121.
